# Profiling School Shooters: Automatic Text-Based Analysis

**DOI:** 10.3389/fpsyt.2015.00086

**Published:** 2015-06-03

**Authors:** Yair Neuman, Dan Assaf, Yochai Cohen, James L. Knoll

**Affiliations:** ^1^Homeland Security Institute and Department of Education, Ben-Gurion University of the Negev, Beer-Sheva, Israel; ^2^Independent Researcher, Petakh-Tikva, Israel; ^3^Gilasio Coding, Tel-Aviv, Israel; ^4^Forensic Psychiatry, State University of New York Upstate Medical Center, Syracuse, NY, USA

**Keywords:** forensic psychiatry, school shooters, automatic text analysis, computational personality, natural language processing

## Abstract

School shooters present a challenge to both forensic psychiatry and law enforcement agencies. The relatively small number of school shooters, their various characteristics, and the lack of in-depth analysis of all of the shooters prior to the shooting add complexity to our understanding of this problem. In this short paper, we introduce a new methodology for automatically profiling school shooters. The methodology involves automatic analysis of texts and the production of several measures relevant for the identification of the shooters. Comparing texts written by 6 school shooters to 6056 texts written by a comparison group of male subjects, we found that the shooters’ texts scored significantly higher on the *Narcissistic* Personality dimension as well as on the *Humilated* and *Revengeful* dimensions. Using a ranking/prioritization procedure, similar to the one used for the automatic identification of sexual predators, we provide support for the validity and relevance of the proposed methodology.

## Introduction

School shooters receive extensive media coverage and create social anxiety that is distinct from other forms of domestic violence. In this context, forensic psychiatrists are often asked to profile the shooters to provide a better understanding of the causes of the atrocities.

The profiling of school shooters should be informative in the sense that it can be used for future screening of potential offenders. Such a screening procedure may identify candidates for (1) in-depth personal diagnosis and (2) preventive steps to be taken by mental health practitioners and law enforcement agencies.

Currently, and for several reasons, there is no consistent diagnosis of school shooters. For instance, it was argued that this psychological diagnosis is often based on symptoms that are shared with other diagnoses (1).

This critique can be illustrated through the case analysis of Seung-Hui Cho who murdered 23 students and faculty members at Virginia Tech on April 16, 2007. Cho was diagnosed as having “paranoid-schizoid dynamics” (2, 3), major depression (1), schizophrenia (4), and selective mutism (5), but as he compared himself to Moses, he can also be diagnosed as suffering from a narcissistic personality disorder (NPD).

The NPD may be considered a central theme of civilian mass murderers in general and school shooters, in particular, as these acts of murder are considered, in some cases, to be acts of revenge (2) that have been theoretically framed as a response to narcissistic injury. For a recent review, and a novel dynamic perspective on revenge used in this paper, see Neuman (6).

In addition to the above mentioned diagnoses of Cho, his documented social detachment may also lead us to diagnose him through the schizoid personality disorder (ScPD), which is characterized by a “pervasive pattern of detachment from social relationships” (7). Indeed, the ScPD is deeply linked to “unbearable and inescapable loneliness” (8) that seems to be a repeating theme evident in the writings of Cho and other shooters. ScPD was found to be correlated with violent behavior (9) and a precursor of violence toward self and/or others (10). This knowledge leads us to add the ScPD aspect to the other diagnoses presented above.

Despite the intensive clinical and forensic work on the subject of mass shooters, the complexity of the phenomena, its negligible proportion in the population, and the difficulty in gaining a psychiatric diagnosis prior to the act of shooting, leads to the current state of affairs where there is no single, clear, agreed upon, and informative clinical diagnosis that can be used for screening and prevention.

In this short paper, we present a novel approach for addressing the challenge of profiling school shooters. Our proposed methodology, validated elsewhere (11), does not pretend to solve the enormous difficulties in profiling and identifying school shooters, but modestly to add another tool to the tool kit of forensic psychiatry and law enforcement agencies. The methodology is based on the automatic extraction of relevant dimensions from texts as explained in the next section.

## The Methodology: Vectorial Semantics and Personality Assessment

The proposed methodology for automatic text analysis has been introduced and validated elsewhere (11), but here is the first time that it is applied to the context of school shooters.

The methodology is based on vector space models of semantics (12). Vector space models of semantics suggest that the meaning of a word(s) can be identified by analyzing words co-occurring with our target word in a given context.

For example, let us assume that we would like to automatically identify the meaning of being *Depressed*. For addressing this challenge, we may search for the adjectives that appear with *Depressed* in the same context.

That is, we search for the adjectives co-located with *Depressed*, which means that we search a huge number of texts for the word “Depressed,” identify the adjectives that appear to the right/left of our target word in a given window (e.g., three words to the right/left of the target word), and identify the words that appear with *Depressed* beyond a certain statistically significant criterion.

Using a corpus of the English language, such as Corpus of Contemporary American English (13), we may find that the adjectives most often co-located with *Depressed* are *Anxious*, *Angry*, *Suicidal*, *Sad*, and *Lonely*.

For the sake of simplicity, let us focus on the two first words: *Anxious* and *Angry*, and assume that in our linguistic corpus *Depressed* appears with *Anxious* six times and with *Angry* three times.

**Figure 1:**
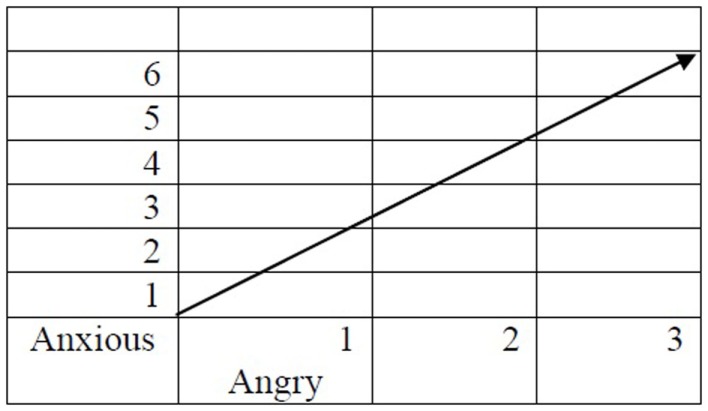
***Depressed* represented in a 2D semantic space**.

At this point, vectorial semantics proposes that we consider *Anxious* and *Angry* as two dimensions defining the meaning of *Depressed*. According to this proposal, the meaning of *Depressed* is represented as a vector in a two-dimensional space defined by *Anxious* and *Angry*, a point graphically presented in Figure 1.

This is of course a simplified representation of *Depressed*. In the above figure, we used only two dimensions to represent the meaning of *Depressed* but we can use more dimensions.

Although we cannot visually imagine a high-dimensional representation of *Depressed*, from a mathematical perspective we can easily build a high-dimensional representation of *Depressed* in which the dimensions are words such as *Anxious*, *Angry*, *Suicidal*, *Sad*, and *Lonely*, and in which the meaning of *Depressed* is represented as a vector in the high-dimensional space.

The point, though, is that by using this approach, we may successfully measure the degree to which the feeling of being *Depressed* is expressed in a text.

For instance, let us assume that we would like to measure the degree of depression in a given text. First, we choose words for defining the vector of *Depressed* such as *Anxious*, *Angry*, *Suicidal*, *Sad*, and *Lonely*.

Next, we take our text and represent it as a vector according to the words which it contains. Now, we can measure the distance between the two vectors. The closer the vectors are, the higher is the expressed degree of depression in the text. This approach can be used for screening for signs of depression in texts (14).

In sum, in measuring the degree to which a certain psychological characteristic appears in a text, the first step in the vectorial semantics approach to personality assessment is to identify words that are the best representatives of a certain personality trait.

These words actually constitute the vector for the analysis. In Neuman and Cohen (11), vectors of various personality dimensions have been constructed by mostly drawing on Millon’s theory of personality (15).

For instance, they have used the word suspicious as one of the important constituents of the Paranoid personality vector.

It must be noted that the vectorial semantics approach to personality assessment that has been scientifically validated (11) using real world data, significantly differs from some of the most popular methods of automatic text analysis for psychological research.

For instance, the lexical approach epitomized by the linguistic inquiry and word count (LIWC) (16) uses a *predefined dictionary* of words that have been categorized by human subjects into different categories (e.g., negative or positive words). Using this approach, we can measure, for example, the percent of positive/negative words in a text by categorizing the words into the negative and positive categories.

The advantage of the vectorial semantics approach used by Neuman and Cohen (11) is that it is not limited to a predefined set of words/dictionary and measures the similarity between two “texts” without being limited by their overlapping words/categories. In any case, the aim of the current paper is not to compare between different approaches to automatic text analysis but to use one approach of “computational personality” in the specific context of school shooters.

The exact methodology will be clarified with regard to the data in the next section.

## The Data

We selected six texts written by school shooters. Most of the texts were downloaded from a site dedicated to the study of mass shooters (https://schoolshooters.info/original-documents). The texts were the manifesto written by Seung-Hui Cho, who murdered 23 students in the Virginia Tech Massacre; the suicide note of de Oliveira, who murdered 11 children in the Rio de Janeiro school shooting; the documents of Pekka Eric Auvinen, who murdered 8 people in the Jokela High School Massacre in Finland; the writings of “Kip” Kinkel, the perpetrator of the Dawson College Shootings, who murdered 4 people; the suicide note of Marc Lépine, who murdered 14 women in the “Montreal Massacre”; and the writings of Luke Woodham, who murdered 3 people.

These texts present a variety of stylistic forms of different lengths, but following the proposal to focus on the “phenomenology” of the perpetrator, as described in their diaries for instance (1), *all of the texts we chose represent the murderer’s first person perspective* before the murders took place.

The idea of focusing on the “phenomenology” of the mental disturbance rather than on symptoms as diagnosed by experts has been already applied, although in a different context, to the identification of depression (14).

We do not aim to present a representative sample of school shooters or their comparison group, as random sampling from the population is irrelevant for practical reasons.

For gaining comparative insights, we used the Blogs Authorship Corpus (17) and selected blogs written by *males* from the age of 15–25, ages approximately overlapping those of the school shooters. Overall, we analyzed the blogs written by 6056 subjects.

## Analysis and Results

### Text processing

First, we use Stanford Part-of-Speech Tagger (18) and extract from the text (i.e., the blog or the text written by the shooter) only three parts of speech categories: nouns, verbs, and adjectives.

From each text, we selected the 10 most frequent nouns, the 10 most frequent verbs, and the 10 most frequent adjectives. Overall, we used 30 words to represent each text as a vector.

Next, we used the vectorial semantics model developed by Turney (19, 20). This model allows us to measure the similarity between words and texts with great accuracy.

We measured the semantic similarity between each of the texts and word vectors representing four personality disorder traits: paranoid personality disorder (PPD), NPD, schizotypal personality disorder (ScPD), and depressivity (DEP).

The words were identified by Neuman and Cohen (11) based on the DSM-V criteria and Millon’s Personality traits. The vectors were as follows:

1. DEP: Sad, lonely, hopeless, worthless
2. PPD: suspicious, hypersensitive, wronged, hostile
3. NPD: arrogant, manipulative, egocentric, insensitive
4. SCHYZO: detached, avoidant, lonely, indifferent

In addition to the four personality vectors mentioned above, and based on Neuman (6) theorization of revenge, we used nine additional word vectors:

1. Hopeless: hopeless, desperate
2. Lonely: lonely, lonesome
3. Helpless: helpless, defenseless
4. Pain: pain, misery, agony
5. Revengeful: revengeful, vengeful, vindictive
6. Chaotic: chaotic, disordered
7. Unsafe: unsafe, insecure
8. Abandoned: abandoned, deserted
9. Humiliated: humiliated, shamed

These vectors aim to represent different facets of *vengeful behavior* that may contribute to the screening procedure.

Overall, we have analyzed 13 vectors; each text was automatically analyzed and its similarity score to the above vectors/variables was determined.

In other words, for each text we analyzed, we automatically produced 13 scores. Based on Neuman and Cohen (11), these scores aim to represent the degree to which a certain personality dimension or behavior appears in the text. The higher the produced score, the greater is the expression of the supposed dimension (e.g., vengefulness) in the text.

For a preliminary analysis, we asked whether there is a difference between the texts written by the shooters and the male bloggers. We compared the scores of the two groups on the dimensions/vectors that we automatically extracted from the texts. Given the negligible number of shooters, we used a non-parametric test – the Mann–Whitney *U* test – to compare the groups with a Monte Carlo simulation of 10,000 samples.

It was found that the school shooters’ texts scored higher on the following dimensions:

1. Revengeful (*U* = 6767, *p* = 0.005),
2. NPD (*U* = 8622, *p* = 0.02), and
3. Humiliated (*U* = 9635, *p* = 0.04)

Given all necessary qualifications and small number of shooters’ texts, we may suggest that the shooters have a significant signature of the NPD as proposed by Knoll (2) and that their texts are significantly different in terms of higher levels of humiliation and revenge.

While these differentiating scores are of no surprise to the forensic psychiatrist, they provide a solid empirical support for our knowledge and intuition based on automatic text analysis. Nevertheless, these results are not the main support of our methodology.

An empirical support for the benefits of our methodology is by testing it in a similar context to the one used for *the automatic identification of sexual predators* (21) in which *a ranked list of suspects is automatically created to prioritize the investigation*. If our proposed methodology can significantly contribute to such a *ranking/prioritization process* then it may gain support for its validity.

### The ranking and prioritization procedure

We followed the logic of the *automatic identification of sexual predators* (21). Our basic assumption was that an expert forensic investigator can identify the texts written by the murderers as texts waving a “red flag.” An effective screening procedure should *significantly reduce the number of texts* the expert should read in order to identify these red flags by ranking the texts according to their manifested dimensions.

For ranking and prioritizing our texts, we used all of the dimension scores described above (e.g., the NPD score) and used them as independent variables for predicting a categorical dependent variable (i.e., shooter or non-shooter).

We used several models for prediction and ranked our texts in descending order according to their predicted probability for being a text written by a school shooter.

Next, we searched for the shooters’ texts by starting from the highest score and counting the number of texts the human expert should read in order to identify all of the texts. This amounts to a strategy of screening for the texts by searching from the top-ranked texts to the bottom and prioritizing the top ranked.

We used three statistical models for the analysis: a binary logistic regression analysis (BLR), a tree classification with CHAID and 10-fold cross-validation procedure (TRE), and K nearest neighbors analysis (KNN) with 10-fold cross-validation.

Table 1 presents the results of our analysis. For each statistical model, we predicted the probability that a text was written by a shooter and using this probability ranked the texts in descending order. The table presents the shooters and their rank according to the model. The right column presents the mean of the three ranks.

**Table 1:** **Results of the ranking procedure**.

BLR	TRE	KNN	Mean of ranks
Cho 1	Pekka 1	Cho 1	Cho 1
Pekka 3	De Oliv 2	Kinkel 47	Kinkel 19
De Oliv 5	Cho 69	Pekka 64	Luke 56
Kinkel 22	Kinkel 79	Luke 161	Lepine 161
Luke 118	Luke 119	De Oliv. 184	Pekka 209
Lepine 227	Lepine 228	Lepine 762	De Oliv. 210

We can see that ranking the texts according to the BLR and the TRE procedure produced the best results. Ranking and prioritization of the texts allow us to identify all of the shooters in 228 steps from the highest ranked text. Using this procedure of prioritization, we have to search approximately 4% of the corpus in order to identify our shooters’ texts.

By averaging the ranks of the texts’ probabilities, we gain the best results: identifying all the shooters’ texts among the top 210 ranked texts, which is approximately *3% of our corpus*.

These results indicate that scoring the texts according to their similarity with our personality/behavioral dimensions and using statistical tools for ranking the texts, allow us to prioritize them in a way that significantly improves the identification of the shooters’ texts.

## Discussion

In this paper, we present a new methodology for profiling texts written by school shooters. Support for the validity of our methodology is given in terms analogous to the identification of sexual predators through ranking and prioritization.

The results of our ranking and prioritization procedure can probably be improved by including more features (e.g., *n*-grams) of text analysis and by experiencing with several methods of machine learning (e.g., SVM). However, we have decided to focus this preliminary work on the ability to identify the texts written by shooters using only psychological dimensions that are theoretically suspected to be associated with school shooters.

From a critical perspective, the difficulties associated with this methodology are clear, although they characterize every automatic text analysis methodology that would have been applied to the same task.

First, the ability to extrapolate from a very small sample of shooters’ texts to future shooters’ texts is limited. The specificity of our sample and its comparative blogs corpus is such that the parameters of the vectors that we have identified in our analysis may be limited to this study only. Moreover, the words chosen for composing the vectors were based on previous theorization but other words could have been selected too, and the impact of word choice on our model is not clear, although experimenting with different vectors created similar results.

On the positive side, the methodology presented in this paper is grounded in clinical knowledge, is generic, and can be designed to examine different psychological theorizations concerning the profiling of and identification of shooters. In fact, the methodology can be used by applying relatively novel and biologically grounded approaches to personality [e.g., Ref. (22, 23)].

In addition, the fact that our methodology is automatic allows us to screen a massive number of texts in a short time. While ethical considerations are inevitable, we can definitely imagine a situation in which parents give the school permission to scan their teenagers’ social media pages under certain limitations.

In this context, using our automatic screening procedure, a qualified psychiatrist or psychologist, who will be trained to work with such a procedure, may automatically get red flag warnings for students whose texts express a high level of potential danger. While this methodology does not provide the magic bullet for identifying potential offenders, and should be cautioussly used given the unknown percentage of false alarms, it clearly presents one pragmatic approach that can be further developed in order to gain better results for screening and prevention. In fact, the problem of “false positives” is an unresolved issue in screening for a small number of potential offenders (24). We do not pretend to solve this problem but introduce a methodology that like other methodologies addressing similar challenges, should be used with the highest degree of sensitivity in a reasonable context of decision making, prices, and alternatives.

## Conflict of Interest Statement

The authors declare that the research was conducted in the absence of any commercial or financial relationships that could be construed as a potential conflict of interest.
